# A case of early autoimmune gastritis with characteristic endoscopic findings

**DOI:** 10.1007/s12328-021-01351-4

**Published:** 2021-02-10

**Authors:** Maiko Kishino, Kenshi Yao, Hiroshi Hashimoto, Hiroki Nitta, Rie Kure, Ayako Yamamoto, Kana Yamamoto, Kouichi Nonaka, Shinichi Nakamura, Katsutoshi Tokushige

**Affiliations:** 1 Institute of Gastroenterology, Department of Internal Medicine, Tokyo Women’s Medical University, 8-1, Kawada-cho, Shinjuku-ku, Tokyo, 162-8666 Japan; 2 Department of Endoscopy, Fukuoka University Chikushi Hospital, Chikushino, Japan; 3 Yokohama Asahi Chuo Sogo Hospital, Health Care Unit, Yokohama, Japan

**Keywords:** Autoimmune gastritis, Type A gastritis, Hashimoto’s disease

## Abstract

Significant atrophic gastritis in the fundic gland region is a well-known endoscopic finding observed in autoimmune gastritis (AIG). The endoscopic features of early AIG have not been reported. Iron deficiency, vitamin B_12_ deficiency, anemia, or neurological symptoms may not be observed in the early stages of AIG, and it may thus be difficult to diagnose early AIG based on clinical findings. We treated a 50-year-old Japanese female whose condition was suspected to be early AIG. The endoscopic findings showed normal gastric pyloric gland mucosa, and diffuse reddened and edematous gastric fundic gland mucosa. Pathologically, local infiltration of lymphocytes and decrease of parietal cells was present in a deep part of the gastric fundic gland mucosa. Blood tests showed that the titer of parietal cell antibody (PCA) was 1:320 and the gastrin level was 820 pg/ml. We determined that the patient had AIG because she also had Hashimoto’s disease, the PCA titer was high, the serum gastrin level was slightly increased, and inflammation was observed only in the gastric body on the endoscopic images. To the best of our knowledge, this is the first case report of endoscopic findings that suggest early AIG, before atrophic changes were observed.

## Introduction

Endoscopic findings are important as a clue to the diagnosis of autoimmune gastritis (AIG). A typical endoscopic finding of AIG is the presentation of significant and severe atrophic gastritis in the fundic gland region, which is also known in Japan as “endoscopic reversed type atrophic gastritis” as reported by Kurokawa et al. [1]. Since the report on Type A gastritis by Strickland et al. was published in 1973, it has been known that an autoimmune-positive result, iron deficiency anemia, pernicious anemia, and hypergastrinemia are observed in typical AIG [2]. AIG was thought to be a rare disease in Japan, but the recent awareness of AIG in Japan has increased, as has the AIG detection rate; this has led to an increased number of reports on AIG [3]. However, there are no reports on the endoscopic features of early AIG, in part because clinical laboratory test results may not be significantly abnormal in the early stages of AIG. Herein, we report the case of our patient who we diagnosed as having early AIG based on endoscopic findings, laboratory findings, and histopathological findings.

## Case report

The patient was a 50-year-old Japanese woman. She had never received eradication therapy for *Helicobacter pylori* (*H. pylori*)*.* She had not used any medications for the treatment of a peptic ulcer such as proton pump inhibitors, or H2-receptor antagonists; nor had she taken any digestive enzyme drugs, non-steroidal anti-inflammatory drugs, or antithrombotic drugs.

She visited our hospital for a detailed examination because abnormal findings had been detected by endoscopy when she underwent a medical check-up. The detected findings in a medical check-up were the diffuse reddened changes in the gastric fundic gland mucosa. She had no symptoms. The results of blood tests performed at the time of her first visit are shown in Table 1. The test result for the presence of the *H. pylori* antibody was negative, and that for thyroid peroxidase (TPO) antibody was positive due to the patient’s Hashimoto’s disease.

**Table 1:** Laboratory data at the first visit to our hospital

Hematology		
White blood cell	4090	/µL
Red blood cell	4.47	× 10^6^/µL
Mean corpuscular volume	90.6	fl
Mean corpuscular Hemoglobin	30.2	pg
Hemoglobin	13.5	g/dl
Hematcrit	40.5	%
Platelet	16.4	× 10^4^/µL
Biochemistry		
Total protein	7	g/dl
Total bilirubin	0.7	mg/dl
Aspartate Aminotransferase	20	U/L
Alanine aminotransferase	14	U/L
Lactate dehydrogenase	185	U/L
Blood urea nitrogen	15.5	mg/dl
Creatinine	0.71	mg/dl
Na	143	mEq/L
K	4.1	mEq/L
Cl	106	mEq/L
Serology		
Thyroid stimulating Hormone	2.22	µU/ml
Free triiodothyronine	2.86	pg/ml
Free thyroxine	1.44	pg/ml
Thyroid peroxidase antibody	96	U/ml

**Fig. 1:**
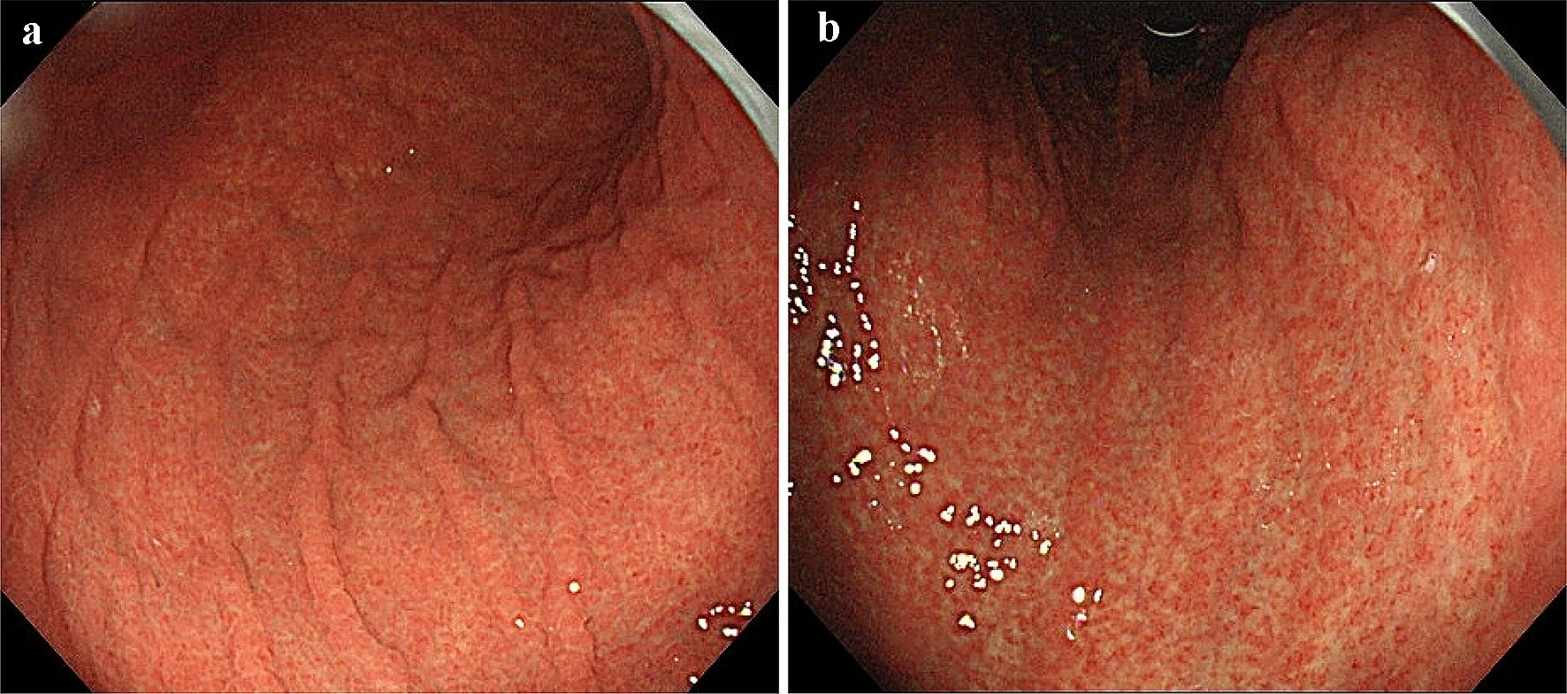
Conventional white-light endoscopic findings. **a** The greater curvature side of the corpus. **b** The lesser curvature side of the corpus. Both the images show diffuse reddened and edematous mucosa without remarkable atrophic change

**Fig. 2:**
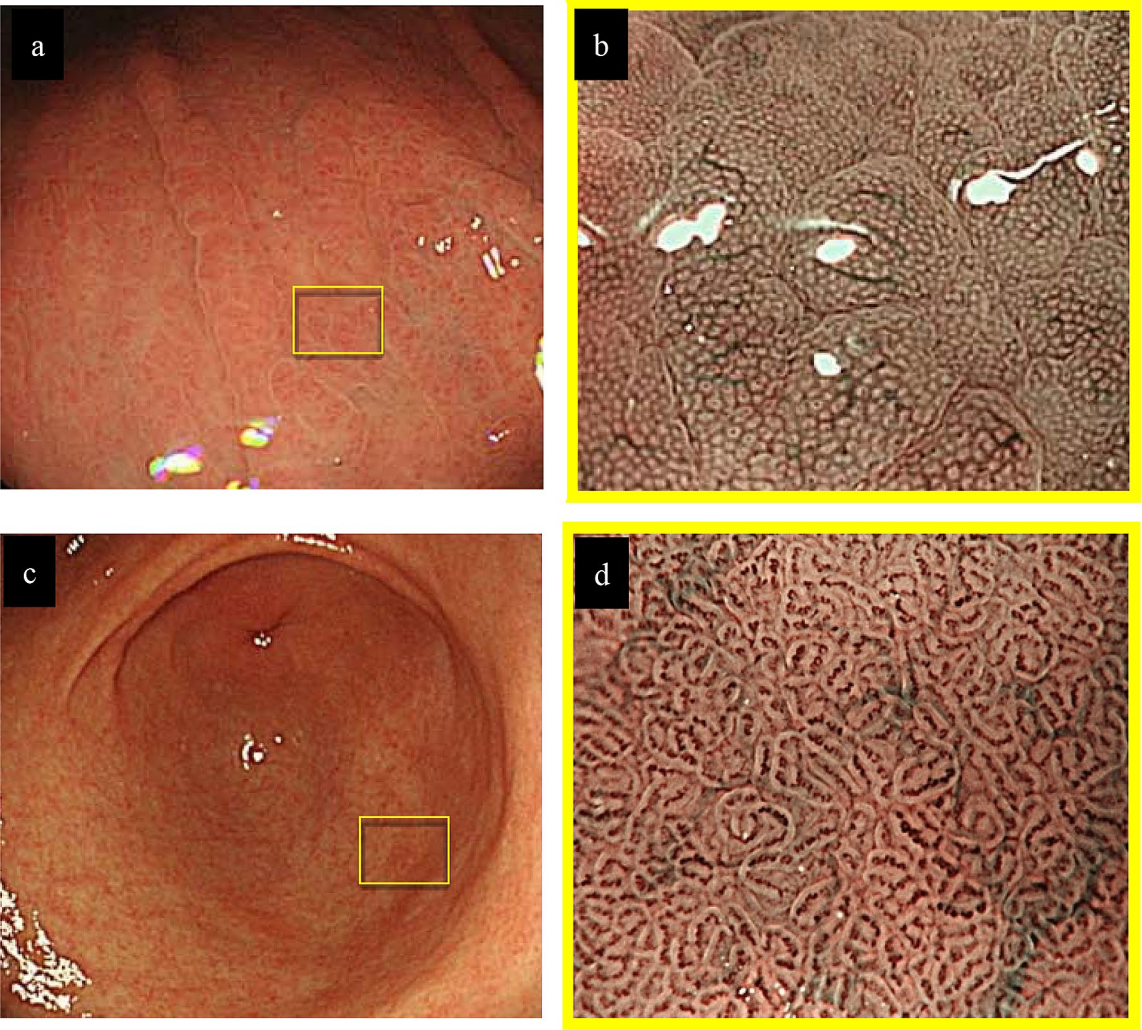
Endoscopic findings. **a** Conventional white-light endoscopic findings of the greater curvature in the middle part of the corpus. **b** The view of the reddened gastric areas by magnifying endoscopy with narrow-band imaging (ME-NBI). **c** Conventional white-light endoscopic findings of the gastric antrum. **d** ME-NBI findings of the pyloric gland mucosa. The microvascular pattern of the fundic gland mucosa showed a dilated SECN with regular honeycomb-like arrangement and collecting venules, and the microsurface pattern depicted a regular oval crypt opening and normal oval MCE (**a**, **b**). Both conventional white-light and ME-NBI findings showed that the gastric pyloric gland mucosa was normal (**c**, **d**). *ME-NBI* magnifying endoscopy with narrow-band imaging, *SECN* subepithelial capillary network, *MCE* marginal crypt epithelium

The endoscopy performed at our hospital revealed reddend and edematous change of the gastric areas extensively in the gastric fundic gland mucosa by conventional white-light imaging (Fig. 1), and not revealed significant atrophic images. As a result of strong extension due to insufflation of a large amount of air during the examination, reddened and edematous change of the gastric areas were observed a mixed finding of the small red ridge and the depressed pale areas. We then observed the microanatomy of the gastric areas by magnifying endoscopy with narrow-band imaging (ME-NBI). The microvascular architecture of the fundic gland mucosa had a regular honeycomb-like subepithelial capillary network (SECN) pattern with collecting venules, and the microsurface pattern showed a regular oval crypt opening and normal marginal crypt epithelium (MCE). These microvascular and microsurface patterns, which were morphologically described in normal fundic gland mucosa by Yao et al. (2008), were well-preserved [4]. Nevertheless, both the SECN and the collecting venules become gradually dilated toward the central part of slightly elevated gastric areas (Fig. 2).

**Fig. 3:**
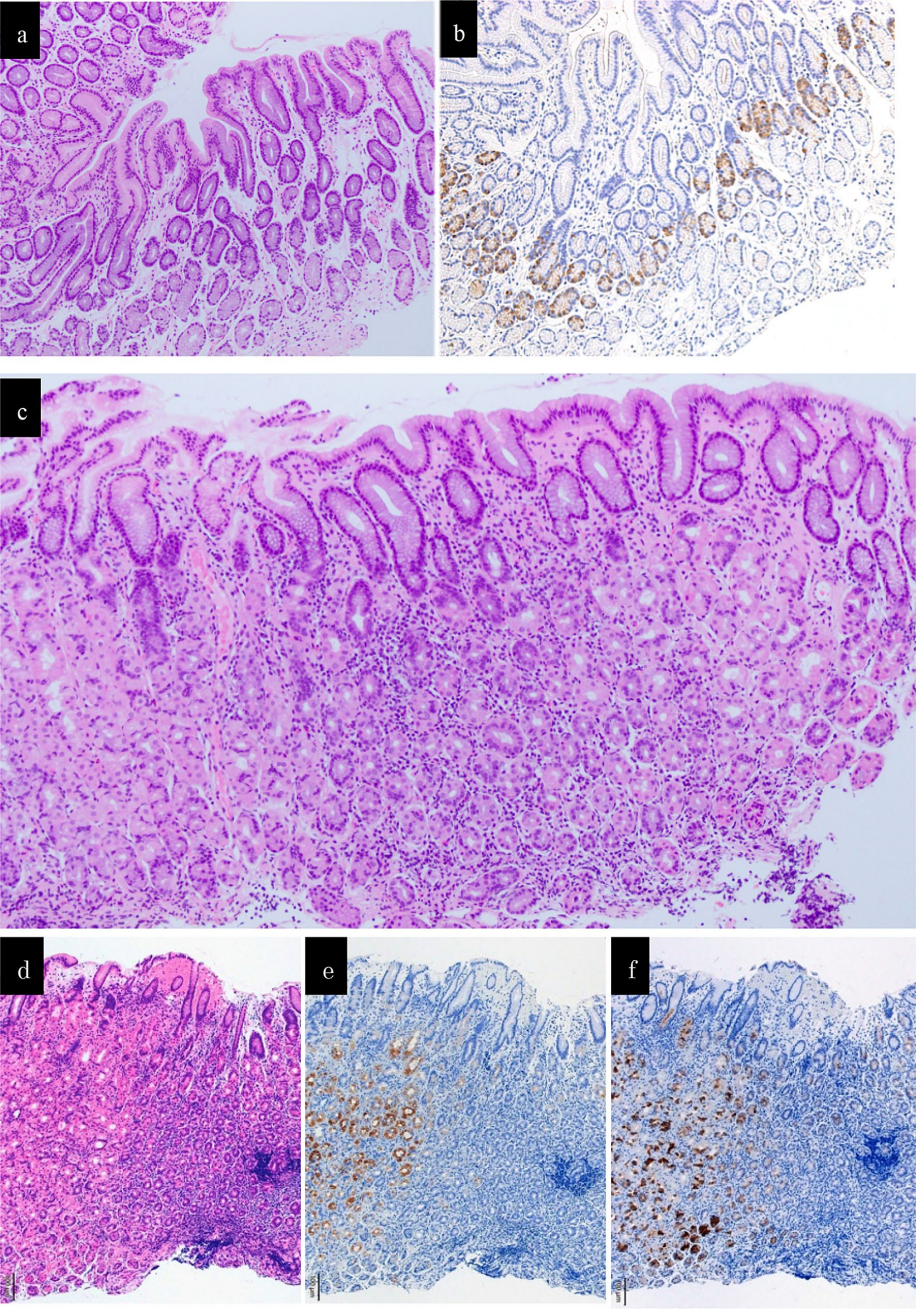
The histopathological findings. **a** The histopathological findings of the specimens biopsied from the lesser curvature in the pyloric gland mucosa did not show either atrophy or intestinal metaplasia (HE staining). **b** The histopathological findings of the specimens biopsied from the lesser curvature in the pyloric gland mucosa showed mild hyperplasia of G-cells (immnostaining for gastrin). **c** The histopathological findings a biopsy specimen obtained from the greater curvature in the middle part of the gastric corpus did not show atrophy, intestinal metaplasia, or decreased parietal cells accompanied with a dense lymphocyte infiltration without neutrophils in the middle-to-deep part of the lamina propria mucosa (HE staining). **d** The finding of HE staining of a biopsy specimen obtained from the greater curvature in the middle part of the gastric corpus. **e** The finding of immunostaining for H ^+^/ K^+^ -ATPase of a biopsy specimen obtained from the greater curvature in the middle part of the gastric corpus. **f** The finding of immunostaining for Pepsinogen I of a biopsy specimen obtained from the greater curvature in the middle part of the gastric corpus. The immunostaining for both H^+^/K^+^-ATPase and Pepsinogen I was negative in the area with a dense lymphocyte infiltration (**d**, **e**, **f**)

No abnormalities in the gastric pyloric gland were observed by conventional endoscopy. In addition, a regular coil-shaped SECN pattern and a regular curved MCE pattern were observed by ME-NBI. Thus, all endoscopic findings showed that the gastric pyloric gland mucosa was normal (Fig. 3).

The histopathological findings of biopsy specimens are shown in Fig. 3. The findings obtained from the pyloric glands region did not show atrophy and intestinal metaplasia. Immunostaining with gastrin showed mild hyperplasia of G-cells. The histopathological findings of a biopsy specimen obtained from the greater curvature in the middle part of the gastric corpus did not show atrophy, intestinal metaplasia, eosinophilic infiltration, noncaseating granulomas, inclusion bodies suggesting viral infection, or decreased parietal cells but did demonstrate a dense lymphocyte infiltration without neutrophils in the middle-to-deep part of the lamina propria mucosa. And immunostaining for H^+^/K^+^-ATPase and Pepsinogen I in this specimen were negative in the area with a dense lymphocyte infiltration.

Although no definitive gastric atrophy was identified on the endoscopic and histopathological findings, we made the diagnosis of early AIG based on the following findings. (1) A diffuse inflammatory change was clearly present in the endoscopic findings limited to the fundic gland part without *H. pylori* infection. (2) Dense lymphocyte infiltration with parietal cell damage was present in the middle-to-deeper part of the lamina propria mucosa in the histopathological findings. (3) The patient had a history of Hashimoto’s disease. In the laboratory examination added after the endoscopy (Table 2), (4) the titer of parietal cell antibody (PCA) was increased to 1:320. (5) Although iron deficiency, vitamin B_12_ deficiency, and a decreased pepsinogen I level were not observed, the gastrin level was high. And no gastrin-producing tumor was found on CT examination. The patient's endoscopic findings might be thus characteristic of AIG at its early stage.

**Table 2:** Laboratory data added after the endoscopic examination

Serology		
*H. pylori* IgG	< 3	U/ml
Parietal cell antibody	320	
Intrinsic factor antibody	–	
Gastrin	820	pg/ml
Pepsinogen I	72.7	ng/ml
Pepsinogen II	17.7	ng/ml
Pepsinogen I/II	4.1	
Biochemistry		
Fe	124	µg/ml
Vitamin B12	437	pg/ml

**Fig. 4:**
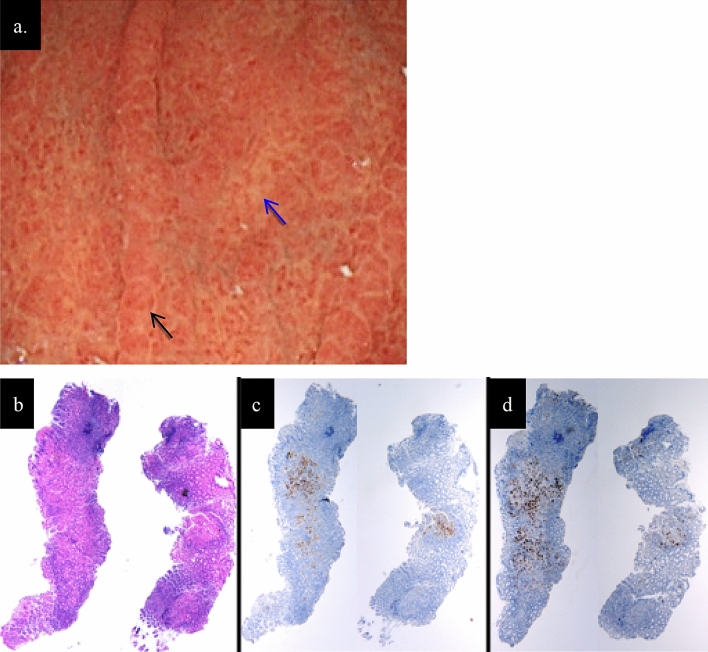
**a** The endoscopic findings of the greater curvature in the middle part of the corpus 1 year later. There were no changes compared to the previous findings. The reddened mucosa (black arrow) and the pale mucosa (blue arrow) where the biopsy was performed were shown in this figure. **b**, **c**, **d** The histopathological findings in biopsied specimens from the reddened mucosa (left specimen) and from the pale mucosa (right specimen) in the greater curvature in the middle part of the corpus. **b** HE staining. **c** Immunostaining for H^+^ / K^+^ -ATPase. **d** Immunostaining for pepsinogen I. The area of lymphocyte infiltration accompanied by parietal cell damage was predominantly present in the pale area (**b**, **c**, **d**)

An year later, the second endoscopy was performed. No significant changes in the gastric fundic gland mucosa and blood tests showed no significant changes in the AIG marker. At the time of the examination, biopsy was performed separately for the small red ridge and the depressed pale area. As a result, the area of lymphocyte infiltration accompanied by parietal cell damage was predominantly present in the pale area (Fig. 4).

## Discussion

AIG has been reported by Strickland and Mackay as a chronic inflammatory disease that yields positive test results for PCA or intrinsic factor antibody, gastric corpus atrophy, gastric acid hyposecretion, and hypergastrinemia [2]. Endoscopists are aware that “reverse atrophy” is a typical endoscopic feature of AIG. In term of ME-NBI findings, Yagi et al. reported that closely arranged small round and oval pits were observed in the atrophic mucosa of AIG [5]. Our patient showed no typical endoscopic findings of AIG. ME-NBI findings also differed from the previously reported by Yagi et al. regarding such endoscopic findings of AIG, the distribution of mucosal inflammatory changes in our patient was the same as that of typical AIG cases, but the detailed endoscopic findings were different. Namely, there was no atrophic change in the gastric fundic gland mucosa; instead, we observed diffuse reddened mucosa and edematous change in the gastric area. When we examined these findings using ME-NBI, it was apparent that both the microvascular pattern and the microsurface pattern in the normal gastric fundic mucosa were well-preserved. Accordingly, we speculated that the reddened mucosa and edematous change were not due to inflammation of the mucosal surface epithelium but rather were due to inflammation in the lamina propria mucosa at a greater depth than the subepithelial part.

In fact, the histopathological findings of the biopsied specimen obtained from the patient’s inflamed mucosa demonstrated that chronic inflammatory cells with parietal cell damage were more densely infiltrated in the middle-to-deep part of the lamina propria mucosa compared to the superficial part. We speculated that the endoscopic findings of reddened mucosa and edema would progress to the typical appearance of atrophic mucosa when the parietal cells were extensively damaged after the inflammation persisted. We thus considered this patient’s endoscopic findings characteristic of the early stage of AIG that had not yet progressed to atrophic gastritis. There has been no prior report of similar endoscopic findings, to the best of our knowledge.

Currently, a definitive diagnosis of AIG is generally made by comprehensive judgment based on endoscopic findings, blood test findings, and histopathological findings. Evidence and data regarding early AIG have been very limited, and this contributes to the significant difficulty in diagnosing early AIG. Torbenson et al. described histopathological features of AIG at an earlier stage, before the parietal cells were completely destroyed [6]. As one of the features, they described damage to fundic glands due to local and diffuse lymphocyte infiltration. In our patient, the damage that we observed in the middle-to-deep part of the fundic gland mucosa is similar to this described feature of early AIG.

We suspected that our patient had parietal cell impairment, which prevents the secretion of gastric acid, because (1) the test result for PCA was positive, (2) hypergastrinemia was observed, (3) mild G-cell hyperplasia in the pyloric gland tissue was observed, and (4) enlarged gastric areas were diffusely observed in the fundic gland region. Although these findings suggested hyposecretion of gastric acid, we did not perform the direct measuring method such as 24-h gastric pH monitoring and could not prove it by serum pepsinogen value alone which is the indirect method. These were the limitation of this report. However, the severity of her parietal cell damage was milder than that observed in typical AIG; and, the fundic glands were well-preserved. Anemia, iron deficiency, and vitamin B_12_ deficiency were not observed. Samloff et al. had already reported on the comparison of PG values in cases classified by histological findings of the gastric mucosa [7]. According to that report, both PG1 and PG2 increased in the "superficial gastritis group", and PG1 decreased in the "atrophic gastritis group" compared with the "normal group" without inflammation and atrophy. Both values of PG1 and PG2 in our case showed an increase.

Accordingly, we speculated that the fundic gland mucosa in this case might be in the pre-stage of the atrophy. In other words, we thus suggested that the stage of AIG in this patient was not at an advanced stage.

Tozzoli et al. reported that in AIG, the titer of PCA decreased over time along with a reduction in parietal cells, which are the target antigens of PCA [8]. We therefore inferred that such a reduction in parietal cells is caused by persistent inflammation, disease progression, and the decreased PCA titer. Given these findings, we determined that the patient, in whom the titer of the antibody was high and the parietal cells were pathologically not reduced, had early AIG.

In conclusion, our patient’s endoscopic findings of diffuse reddened and edematous change in the gastric fundic gland mucosa and the histological findings of focal infiltration of chronic inflammatory cells into the middle-to-deeper mucosal layer might be features of early AIG. In the future, it is necessary to accumulate similar cases and clarify the natural course of AIG.
